# The Universities and Parliament

**Published:** 1918-11-30

**Authors:** 


### The Hospital

The Workers' Newspaper of Administrative Medicine and Institutional Life, Administration, National Insurance and Health.

No. 1695, Vol. LXV. SATURDAY, NOVEMBER 30, 1918. PRICE TWOPENCE

### THE UNIVERSITIES AND PARLIAMENT.

Only a few years ago the representation of the Universities in Parliament seemed a lost cause. The political axe was laid at the root of the tree and the threat was uttered in menacing and confident accents. But threatened institutions are proverbially long livers, and by the chances and changes of time we see to-day, not only the persistence of the University seats but also an increase in their number. The impending doom of an earlier date has been replaced by the added vitality of a new franchise law. As everybody knows, this position is one of the results of a scheme of compromise agreed to by the various political parties and afterwards accepted by the Legislature. The University politicians are able therefore to continue to exercise the franchise in their academic constituencies as well as in their several geographical areas, and the impending election finds them with unrestrained privileges. It is more than probable that the manner in which they use these privileges will be closely watched. The opponents of the system of special University representation have, it is true, consented to cease their antagonism, at least until the next Franchise Bill. But their consent has been obtained as the result of a bargain, and there is no reason to believe that it implies a change of opinion. The University graduate still appears to them as a "plural voter," and indeed he is relatively more conspicuous in this respect in consequence of a reduction of kindred opportunities among the general body of the electors. He stands almost alone as an outrage on the simple and inviting principle which summarises itself in the formula "one man, one vote."

In these circumstances it is impossible to doubt that the actual working in practice of the University franchise will be jealously watched. This time it has escaped condemnation by a sort of happy bargain. But another time of trial will certainly arrive and the present acquittal is no guarantee for the future. The arrangement may therefore be said to be on its trial at the forthcoming election and in due course the result will come under review. This is one reason why the supporters of the plan should exert themselves to ensure its prosperity. Still further, it must be allowed that the double vote appears as an exception to the general rule, and that in the very nature of things it stands on its defence. What is the common lot may easily pass without comment, but a special privilege challenges observation. The University vote, in short, has to justify its existence, and the judgment in the long run will depend upon the actual results of the scheme in practice. If these are purely and solely political in the narrow party sense of the term, it is inevitable that when the opportunity occurs the hostile critics will assert themselves and will press their advantage. For, let it be freely admitted, if all the graduates can show for their special opportunity is the return of members who in no fashion differ from members elected by non-academic constituencies, it will reasonably be asked why the graduate should have two votes when each of his fellow-citizens has only one, and a justification of such an arrangement will be sadly to seek. The alternative obviously is the election of members who, whatever their party designation, have attained a definite level of professional distinction and who are able to contribute from a personal store some special form of knowledge or of counsel to the Parliamentary debates. The value of members of this type hardly anyone questions. Yet the working of the party machine in the ordinary constituencies generally fails to provide them. Here, then, is the chance for the Universities. If they prove themselves capable of accepting it the academic vote will have a good defence when the next time of testing arrives. But if, on the contrary, the vote fails to contribute to the House of Commons some element of distinction it will hardly escape the zeal of the reformer. Circumstances have provided it with a new lease of life. Whether there will be a renewal or not depends on the fashion in which the graduates exercise their special privileges, and the next few weeks will give in some measure the answer to this inquiry.

